# Profile of drug users in the residential treatment centers of Tehran, Iran

**DOI:** 10.15171/hpp.2019.34

**Published:** 2019-08-06

**Authors:** Hossein Akbari, Mohsen Roshanpajouh, Keramat Nourijelyani, Mohammad-Ali Mansournia, Afarin Rahimi-Movaghar, Kamran Yazdani

**Affiliations:** ^1^Department of Epidemiology and Biostatistics, School of Public Health, Tehran University of Medical Sciences, Tehran, Iran; ^2^Addiction Researches Department, School of Behavioral Sciences and Mental Health (Tehran Psychiatry Institute), Iran University of Medical Sciences, Tehran, Iran; ^3^Iranian National Center for Addiction Studies (INCAS), Tehran University of Medical Sciences, Tehran, Iran

**Keywords:** Substance abuse treatment centers, Substance-related disorders, Substance abuse, intravenous

## Abstract

**Background: ** The current study aimed at determining the pattern of drug dependence and its related factors in mid-term residential treatment centers (MTRCs).

**Methods: ** The current cross-sectional study was conducted on all drug dependent people residing in MTRCs of Tehran, Iran, who were voluntarily seeking treatment from April to August, 2018. Required data were collected through face-to-face interviews. Drug dependence was categorized into four groups: soft opioids, hard opioids, methamphetamine, and combination of hard opioids and methamphetamine. The association of potential risk factors with drug dependence was measured using chi-square test and multinomial logistic regression.

**Results: ** Out of 1868 participants in the current study, 97% were male. Mean age (SD) of the participants was 38.1 (9.9). The different types of drug dependence were hard opioids (43.0%),soft opioids (29.5%), methamphetamine (15.4%), and a combination of hard opioids and methamphetamine (12.1%). The prevalence of injecting drug use was 2.7%. In comparison with the reference group (soft opioids), being un-married and unemployment, significantly increased the risk of using the three groups of drugs (odds ratios [ORs]: 1.5-3.34, P values: <0.001-0.033).Age ≥30 years at the initiation of drug use, and using current drug for more than 10 years, significantly increased the risk of using hard opioids and a combination of hard opioids and methamphetamine (ORs: 1.65-2.31, P values: <0.001-0.030). Age ≥50 years significantly decreased the risk of using the three groups of drugs (ORs: 0.21-0.43, P≤0.001).

**Conclusion:** Different pattern of drug dependence found in MTRCs, they were mostly hard opioid users, lower injecting drug use and higher mean of age.

## Introduction

Drug dependence and abuse are among the most important mental and behavioral disorders in the community and are considered as one of the most important risk factors for communicable diseases, non-communicable diseases, road accidents, and the related mortalities.^1,2^ According to the United Nations Office on Drugs and Crime (UNODC), in 2016 more than 31 million people in the world had drug use disorders with a mortality estimate of 450 000 deaths, 16 750 of which were directly due to overdose. More than 76% of these mortalities were due to opioids. Also, in that year, about 22 million lives were lost due to premature death or disability due to substance abuse disorders.^3,4^ Therefore, in the 2030 Agenda for Sustainable Development and to end epidemics of AIDS, tuberculosis, and other contagious diseases, special attention is paid to the prevention and treatment of substance abuse.^5^

A survey of disease burden in Iran in 2010 suggested that the mortality rate due to substance abuse disorders per 100 000 people rose from 4.6 to 7.9 in males and from 0.26 to 0.72 in females from 1990 to 2010.^6,7^ In a national survey conducted in 2011, the prevalence of drug dependence in the general population was estimated 2.1%.^8^ Opioids including opium, heroin, cannabis, and methamphetamine are the most commonly used illicit drugs in Iran.^9^

In Iran, different types of centers offer drug treatment and harm reduction services, each of which attracts a different pattern of clients. Outpatient drug treatment and the medium-term residential treatment centers (MTRCs) are the most commonly known types. Inpatient drug treatment services, therapeutic communities, and drop-in centers providing harm reduction services are used by a relatively smaller number of drug users. These centers are accredited and supervised by health or social welfare sectors. In addition, there are residential centers to treat the individuals that are arrested; such centers are under the supervision of law enforcement agencies. Self-help groups such as Narcotics Anonymous are also quite active and provide support and treatment to a large number of drug users.

Although many studies are so far conducted in Iran to understand the patterns of drug use and dependence, firstly, the patterns of drug use and continued drug consumption leading to dependence are dramatically changed in the country in the past two decades, especially in the last five years, which highlights the necessity of monitoring the situation; secondly, few studies are performed regarding MTRCs clients. Also, due to the type of services provided in such centers, there may be a different pattern of drug use and dependence in MTRCs compared to other centers and facilities. Therefore, the current study aimed at determining the patterns of drug use and dependence in MTRCs of Tehran Province and investigating the related factors in 2018.

## Materials and Methods

### Study design and participants

The current descriptive-analytical and cross-sectional study was conducted on 1914 subjects that voluntarily attended MTRCs affiliated to Iran’s Welfare Organization in Tehran province for drug dependence treatment from 28 April to 22 August 2018. Tehran province, including Tehran (the capital of Iran), has a population of about 13.5 million, accounting for more than 16% of the total population of the country.

### Medium-term residential treatment centers

MTRCs provide health care for a period of 28 to 90 days. In the centers, drug addicts often have a longer history of drug dependence and the focus is on supporting residents, training them the coping skills, and preventing them from relapse. In these centers, a 12-step program is provided to the residents and they are encouraged to participate in self-help groups after being discharged from these centers.^10^

The data collection was performed anonymously and the participants were ensured that participation or lack of participation in the study did not affect the provided services or the treatment process.

### Data collection

In order to collect data, a checklist was designed. For this purpose, first, resources in electronic databases were searched. Then, the factors influencing drug dependence were extracted, and subsequently, the checklist was edited and finalized by experts. The checklist was completed by expert interviewers through face-to-face interviews in MTRCs. For the qualitative control of data collection, a training course was held in three sessions and one observer continuously monitored the data collection process.

### Variables

In the current study, the following variables were recorded: gender (female/male), age (<30; 30-39; 40-49; ≥50 years), marital status (unmarried/married), level of education (illiterate/primary/secondary/high school/university), occupation (unemployed/employed), place of residence (Tehran province/other provinces), self-reported subjective social status (low/middle/high), family history of substance use (no/yes/unknown), roommate status (alone/ with somebody else), age at initiation of drug use (<20 / 20-29 / ≥30 years), the number of times referred for rehabilitation (admission times count), the funding sources for the treatment (personal/family or relatives/governmental expenses), route of drug use (smoking/ injection/ inhalation/oral use/ more than one route), the reason for referring to MTRCs (self-preference/ employer compulsion/ family compulsion/ other reasons), the reason for referral (self-preference/ family or friend suggestion/ physician recommendation/ other reasons), the type of substance used (soft opioids/ hard opioids/ methamphetamine/ methamphetamine + hard opioids), and the length of time the current drug was used (<2.9 / 3–9.9 / ≥10 years).

Drug dependence was classified into four categories as follows: (*a*) Soft opioids including opium (*shireh;* the condensed extract of remnants of smoked opium), morphine, methadone and tramadol, (*b*) hard opioids including heroin and crystalized form of heroin,^11^ (*c*) methamphetamine, and (*d*) hard opioids and methamphetamine. Subjects with simultaneous dependence on soft and hard opioids were assigned to hard opioids group and the ones with simultaneous dependence on soft opioids and methamphetamine were allocated to the methamphetamine group.

Based on the recommendations by Finch et al, to determine the socioeconomic status of the participants, the subjective social status (SSS) assessment was used rather than the socioeconomic status (SES) assessment. In this approach, the respondent compares the residents of Tehran and rates his/her socioeconomic status as low, medium to low, medium, medium to high, and high.^12^ In order to resolve the sparse data bias, this variable was classified into the three levels of low (low and lower than average), middle, and high (above average and high).

The participants’ age was classified as <29, 30-39, 40-49, and >50 years, and age at the initiation of the current drug use was classified into the three classes of <19, 20-29, and >30 years. The duration of using the recent substance was classified into three categories of <3, 3-10, and ≥10 years.

### Statistical analysis

In order to describe the statistical data, frequency and percentage were used based on the type of variable. To measure the relationship between each of the factors influencing drug dependence (four categories of drugs used), chi-square test was used, and multiple multinomial logistic regression was used to investigate the confounding and simultaneous effects of independent variables on the drug dependence. In this model, soft opioid users were considered as the reference group. Also, for the age variable, the age group of 30-39 years was considered as the reference group as it was the most frequent. In order to best fit the data with the multiple model, the variables with *P* value of ≤0.2 in univariate analysis were entered into the model by forward stepwise selection, and by comparison with the likelihood ratio, the model with the least number of parameters and the best fit was selected*. P* value <0.05 was set as the significance level. All the analytical steps were performed with IBM SPSS Statistics 22.0 (SPSS for Windows Inc., Chicago, Illinois).

## Results

In the current study, of the 2150 eligible people, 1930 patients from 54 MTRCs were enrolled from 28 April to 28 August 2018 (response rate: 89%). Overall, 23 cases were excluded since they did not complete the checklist correctly or completed it with a specific pattern, and 39 were excluded as the main substance used by them did not fit the four categories of substances (cannabis and marijuana). Eventually, data on 1868 people was analyzed.

The baseline characteristics and clinical information of the participants are shown in Table 1. According to this table, the most common age group was 30 to 39 years (range = 50, median = 37.0, mean ± SD = 38.05 ± 10.0). Most cases consumed their current substance after the age of 30 years (range = 55, median = 28.0) and consumed this substance for more than 10 years (range = 49.6, median = 20.0).

The current study also showed that 50% of the studied population began their first substance under the age of 20 (range = 61, median = 20.0, mean ± SD = 23.2 ± 8.5). The most common types of drug dependence and injectable drug use distributions are shown in Table 2. The most common types of drug dependence were respectively hard opioids, soft opioids, methamphetamine, and both methamphetamine and hard opioids. Also, in 2.7% of the cases, the current drug used was in the injectable form, most of whom belonged to the soft opioids group, and the least proportion was related to the combination of hard opioids and methamphetamine group. Also, 3.5% of the subjects had a history of drug injection during their lifetime.

In univariate analysis, variables such as age, marital status, age at initiation of the current drug use, duration of the current drug use, psychosocial status, occupation, family history of substance use, and residence status were significantly associated with the type of drug used. As shown in Table 1, in the age group of >50 years, the most commonly used type of drug was soft opioids and in other age groups, it was hard opioids. Most married subjects consumed soft opioids and most unmarried participants used hard opioids, and using more than one substance was observed most commonly among unemployed subjects.

Table 3 shows the final multinomial logiscti regression model in which variables such as age, marital status, psychosocial status, occupational status, age at the initiation of the current drug use, and the duration of consumption were associated with the type of substance used. As noted in Table 3, in the age group of >40 years, the odds of consuming all kinds of hard opioids, methamphetamine, and more than one type of substance were lower compared to the age group of 30-39 years, and the highest odds of taking more than one substance was seen in the age group of <29 years.

Unmarried subjects compared to the married ones and unemployed subjects compared to the employed ones had higher odds of consuming hard opioids, methamphetamine, and combination of hard opioids and methamphetamine. Individuals with a medium SSS had higher odds of using hard opioids and the ones with a high SSS had higher odds of methamphetamine abuse. The odds of using hard opioids and more than one type of substance in people that initiated the current substance use above the age of 30 were higher than those of the other age groups. Also, subjects with more than 10 years of addiction to the current drug were more likely to use a combination of hard opioids and methamphetamine.

## Discussion

The current study aimed at determining drug dependence patterns in MTRCs of Tehran Province, Iran and investigating the factors affecting them in 2018. The current study showed that the most common types of drug dependence in Tehran’s residential centers were hard opioids, soft opioids, methamphetamine, and a combination of hard opioids and methamphetamine, respectively. However, according to the Iranian National Mental Health Survey (IranMHS) conducted in 2011 on the general population of Iran aged 15-64, opioids, especially opium, which is a soft opioid, were the commonest ellicit drug and drug dependence in the country.^8^

In addition to Iran, opium is traditionally consumed in countries such as Afghanistan, Pakistan, India, Myanmar, Laos, and China.^13^ National studies on drug users in 1990 and 2007 in three groups of homeless addicts, prisoners, and treatment center clients showed that opium use ranged 43.9% to 73%, heroin use 20.3% to 39.4%, and crack (of heroin) 0% to 28.1% in such groups.^14,15^ Comparison of these data with the findings of the present study suggested that the consumption of soft opioids was lower in MTRCs’ clients and the proportion of hard opioid users was approximately equal to that of the RSA 2007 study.

Amphetamine substances are the second most commonly consumed substances in the world after cannabis.^3^ According to IranMHS, the one-year prevalence of amphetamines and methamphetamine consumption was 1% and 0.7%, respectively.^16^ A review study estimated the prevalence of methamphetamine use among school and university students in Iran over a year before the study from 0.2% to 10.5%.^17^ According to RSA 2007, 3.4% of addicts referring to treatment centers were the methamphetamine users.^14^ In the present study, 15.4% of the subjects had methamphetamine dependence and 12.1% consumed methamphetamine plus hard opioids, indicating the increased dependence on methamphetamine in patients attending MTRCs in 2018.

The current study showed that the mean age of the subjects with drug dependence in MTRCs of Tehran Province was 38.1 ± 9.9 years and the age group of 30-39 years constituted the majority of the subjects (38.5%). In IranMHS study, majority of the subjects with drug dependence belonged to the age group of 20-39 years.^18^ Also, the mean age of drug users in the current study was 4.9 years higher than that of the RSA 2007 study and four years higher than that of a study performed in Birjand, Iran.^18,19^ In a systematic review study including more than 13 000 drug users, the mean age was 32.4 years.^20^

With advancing age, the risk of consuming all types of hard opioids and methamphetamines reduced, and the risk of taking hard opioids and methamphetamine was higher in the age group of <30 years. In other studies, a relationship was observed between age and type of drug abuse and drug dependence.^19,21,22^ The difference in drug use patterns among different age groups can be due to the recent aging trend in Iran or the presence of age-period-cohort effect, since soft opioids are more popular in higher age groups due to the higher prevalence of such drugs in the past.

The mean age at the initiation of the first drug use was 23.2 ± 8.5 years in the current study that was roughly equivalent to the RSA 2007 and the study by Karrari et al and higher than the report by Goodarzi et al on patients referring to the outpatient treatment centers (methadone maintenance treatment [MMT] clinics) in Shiraz, Iran.^19,23^ The mean age at the initiation of the current drug use was 28.7 ± 10.2 years, and the mean length of the current drug use was 8.6 ± 7.8 years. Multivariate analysis indicated that age >30 years at the initiation of drug use and drug use duration of >10 years were significant risk factors for dependence on various types of hard opioids, methamphetamine, and their combination.

In the current study, 17.6% of the subjects were unemployed that was below the unemployment rate reported in the RSA 2007 and the study by Goodarzi.^14,23^ Also, 47% of the cases were married, which was roughly equivalent to the percentage reported in the study by Goodarzi et al.^23^ The current study results showed that employment and marital status were significant factors in determining the type of drug dependence; hence, employed and married people showed a lower risk of using hard opioids, methamphetamine, or their combination, which was similar to the findings reported by Karrari et al.^19^ In the IranMHS study, unemployment was also identified as a risk factor for illicit drug use.^8^ The impact of these two factors can indicate the important effect of social factors of substance abuse and dependence, which should be considered by decision makers.

In the current study, SSS was medium in about 47% of the cases. SSS is an influential variable in the type of substance dependence, since the subjects that categorized themselves as middle class were at a higher risk for dependence on hard opioids and methamphetamine. In the IranMHS study, low socioeconomic status was reported as a risk factor for illicit drug use, and methamphetamine consumption was greater in the ones with an average socioeconomic status.^16^ Regarding the relationship between the type of substance dependence and SSS, issue of temporality should be considered between the two factors, which cannot be judged due to the design of the study.

In most studies in Iran, female cases constituted about 5%-10% of drug users.^18,19,24,25^ Gender (female) is introduced as a factor affecting less referrals to receive treatment and hard reduction services.^26,27^ In the current study, the proportion of methamphetamine consumption and simultaneous use of hard opioids and methamphetamine in females was higher than those of males, but this difference was not statistically significant, which can be due to the limited number of female subjects that voluntarily attended MTRCs. The lack of referral of female drug users to receive addiction treatment services can be due to various factors, including the stigma of this issue among female subjects,^28^ highlighting the need for further studies in this field.

In the current study, education level of 63% of people was up to high-school and a low percentage was illiterate or had academic education, confirming the findings of Karrari et al. In the IranMHS study, the majority of drug users had primary or secondary education, and in the RSA 2007 study, illiteracy rate was 2.1%, and 56% had up to high school education.^8,14,19^ The level of education in the study by Karrari et al was reported as a factor influencing the type of drug abuse, but in the present study, there was no relationship between the level of education and the type of used drug.

In the current study, 2.7% of the subjects were injecting drug users. Meanwhile, in the RSA 1999, more than 16% of the subjects had the experience of drug injection in the past month, and according to the RSA 2007 study, 21.3% of the subjects were the active injecting drug users.^14,15^ Several in-press reports are available on the reduction of injection drug use in Iran. According to the expansion of harm reduction programs in Iran and raising awareness in the society regarding the risks of overdose and the development of blood-borne diseases through injection drug use,^29-31^ and despite the under-reporting of high-risk behaviors among drug users, there was a significant decline in injection drug use in Iran.

### Limitations

The current study was performed on volunteers attending Tehran MTRCs to quit drug dependence and did not include the ones compulsorily referred to such centers for rehabilitation, and the findings of the study may not be generalizable to clients using other methods including non-residential outpatient treatments such as MMT centers. Other limitations of the current study were the small sample size and the multiple levels of independent variables. In order to resolve this issue, it was attempted to merge the categories and reduce the number of parameters in the regression model.

## Conclusion

Results of the current study showed that profiles of the three groups of hard opioid, methamphetamine, and the combination of hard opioids and methamphetamine users were similar, but soft opioid users, which accounted for about one-third of the subjects, basically had different profiles compared to the other three groups. They had a higher age and shorter drug use duration and were more likely to be employed and married. However, two decades ago, the main drug use pattern was soft opioids use in Iran, and as a result, the profiles of addicts were similar. Addicts’ profile changed with the emergence of new drugs. Notwithstanding the changes that indicate an increase in the state of addiction in Iran, two other indicators indicate the improvement; one is the decrease in injection drug use and the other is the increase in the mean age of drug addicts. Confirmation of the above issues requires more extensive studies both from sampling site and geographical region.

## Ethical approval

The current study protocol was approved by the Ethics Committee of the School of Public Health, Tehran University of Medical Sciences, on 08 October 2017, with the ethical code of IR.TUMS.SPH.REC.1396.3649. Also, all the individuals willing to participate in the study provided informed oral consent.

## Competing interests

The authors declare that they have no competing interests.

## Funding

The study received no financial support.

## Authors’ contributions

HA, KY, ARM: study conceptualization, statistical analysis, results interpretation, drafting and revising of the manuscript; MM: study conceptualization and statistical analysis, results interpretation; MR: study conceptualization and data collection; KN: statistical analysis, results interpretation and revising of the manuscript. All authors approved the final manuscript.

## Acknowledgments

The current study was conducted as part of a PhD dissertation in Epidemiology, entitled “Indirect estimation of population size of drug users in MTRCs of Tehran Province based on zero truncated count data” submitted to Tehran University of Medical Sciences. Authors wish to thank all people that collaborated in the study including Mr. Dolati, the Managing Director of Residential Centers of Tehran and his colleagues.

**Table 1:** The baseline characteristics of the participants and univariate analysis of the factors affecting the type of drug dependence (chi-square test)

**Variable**		**Soft opioid**	**Hard opioid**	**Methamphetamine**	**Methamphetamine + Hard opioid**	**Total**	***P*** ** value**
Sex	Male	537 (29.6)	783 (43.1)	279 (15.4)	216 (11.9)	1815 (100)	0.679
	Female	14 (26.4)	21 (39.6)	9 (17.0)	9 (17.0)	53 (100)	
Age	<30	92 (24.1)	148 (38.7)	70 (18.3)	72 (18.8)	382 (100)	<0.001
	30–39	176 (24.5)	326 (45.4)	129 (18.0)	87 (12.1)	718 (100)	
	40–49	138 (30.7)	201 (44.7)	67 (14.9)	44 (9.8)	450 (100)	
	≥50	143 (45.4)	128 (40.6)	22 (7.0)	22 (7.0)	315 (100)	
Marital status	Unmarried	229 (23.3)	431 (43.9)	156 (15.9)	166 (16.9)	982 (100)	<0.001
	Married	320 (36.4)	369 (41.9)	132 (15.0)	59 (6.7)	880 (100)	
Level of education	Illiterate	51 (30.9)	78 (47.3)	20 (12.1)	16 (9.7)	165 (100)	0.527
	Primary	95 (28.8)	135 (40.9)	56 (17.0)	44 (13.3)	330 (100)	
	Secondary	210 (31.1)	290 (42.9)	108 (16.0)	68 (10.1)	676 (100)	
	High School	147 (28.8)	219 (42.9)	72 (14.1)	73 (14.3)	511 (100)	
	University	47 (25.8)	82 (45.1)	30 (16.5)	23 (12.6)	182 (100)	
Occupation	Unemployed	65 (19.7)	151 (45.8)	46 (13.9)	68 (20.6)	330 (100)	<0.001
	Employed	486 (31.6)	652 (42.4)	242 (15.7)	157 (10.2)	1537 (100)	
Residence	Tehran Province	402 (29.6)	568 (41.9)	210 (15.5)	176 (13.0)	1365 (100)	0.187
	Other provinces	149 (29.3)	223 (45.9)	77 (15.2)	49 (9.6)	508 (100)	
Subjective social status	Low	249 (32.9)	304 (40.2)	107 (14.2)	96 (12.7)	756 (100)	0.029
	Middle	226 (25.8)	400 (45.6)	143 (16.3)	108 (12.3)	877 (100)	
	High	76 (32.3)	100 (42.6)	38 (16.2)	21 (8.9)	235 (100)	
Family history of substance use	Yes	15 (27.3)	20 (36.4)	17 (30.9)	3 (5.5)	55 (100)	<0.001
	No	418 (31.2)	602 (44.9)	183 (13.6)	138 (10.3)	1341 (100)	
	Unaware	118 (25.0)	182 (38.6)	88 (18.6)	84 (17.8)	472 (100)	
Roommate status	Alone	122 (30.6)	172 (43.1)	48 (12.0)	57 (14.3)	399 (100)	0.107
	With somebody else	429 (29.3)	629 (42.9)	240 (16.4)	1666 (11.5)	1466 (100)	
Age of initiation of current drug	<20	80 (26.7)	114 (38.0)	62 (20.7)	44 (14.7)	300 (100)	0.001
	20–29	215 (30.2)	288 (40.4)	110 (15.4)	99 (13.9)	712 (100)	
	≥ 30	251 (30.6)	381 (46.5)	108 (13.2)	80 (9.8)	820 (100)	
Duration of the current drug use	< 3.0	180 (31.7)	250 (44.1)	88 (15.5)	49 (8.6)	567 (100)	0.001
	3.0–9.9	148 (25.2)	245 (41.7)	96 (16.4)	98 (16.7)	587 (100)	
	≥10	202 (30.1)	293 (43.7)	101 (15.1)	74 (11.0)	670 (100)	
Total		551 (29.5)	804 (43.0%)	288 (15.4%)	225 (12.1%)	1868 (100)	

**Table 2:** Distribution of the main drug used and injecting drug use in medium-term residential centers in Tehran province

**Drug type**		**No. (%)**	**IDU (%)**
Soft opioids n=551 (29.5%)	Opium	417 (75.7)	17 (3.1)
	Shireh-Opium	50 (9.1)	
	Morphine	46 (8.3)	
	Tramadol	13 (2.4)	
	Methadone	25 (4.5)	
Hard opioids n=804 (43.0%)	Heroin	729 (90.7)	24 (3.0)
	Crack *(of heroin)*	4 (0.5)	
	Hard + Soft opioids	71(8.8)	
Methamphetamine n=288 (15.4%)	Methamphetamine	257 (89.2)	7 (1.7)
	Soft opioids + Methamphetamine	31 (10.8)	
Methamphetamine + Hard opioids n= 225 (12.1%)		255 (100)	3 (1.3)
Total		1868 (100)	51 (2.7)

Abbreviation: IDU, injecting drug use. Sixty participants were using drugs other than the mentioned four categories, including one person who was using cocaine and 59 who were using cannabis. In these cases, 13 individuals used the main drug along with soft opioids, 20 with hard opioids, 5 with methamphetamine, and 21 with combination of methamphetamine and hard opioids.

**Table 3:** Results of multinomial logistic regression of the factors affecting the use of opioid and amphetamine drugs^a^

**Variable**	**Hard opioid, n= 764**		**Methamphetamine, n= 277**		**Methamphetamine + Hard opioid** **n= 219**	
	**Adjusted OR (95%CI)**	***P*** ** value**	**Adjusted OR (95%CI)**	***P*** ** value**	**Adjusted OR (95% CI)**	***P*** ** value**
Age						
<30	1.16 (0.80 to 1.68)	0.444	1.23 (0.78 to 1.94)	0.386	1.81 (1.11 to 2.95)	0.018
30–39	1.00		1.00		1.00	
40–49	0.74 (0.54 to 1.02)	0.062	0.71 (0.48 to 1.06)	0.098	0.64 (0.40 to 1.03)	0.066
≥50	0.43 (0.31 to 0.60)	<0.001	0.21 (0.12 to 0.36)	<0.001	0.30 (0.17 to 0.54)	<0.001
Marital status						
Married	1.00		1.00		1.00	
Unmarried	1.62 (1.28 to 2.06)	<0.001	1.50 (1.10 to 2.05)	0.011	3.34 (2.31 to 4.85)	<0.001
Occupation						
Employed	1.00		1.00		1.00	
Unemployed	1.82 (1.30 to 2.54)	0.001	1.60 (1.04 to 2.47)	0.033	3.07 (2.02 to 4.67)	<0.001
Subjective social status						
Low	1.00		1.00		1.00	
Middle	1.50 (1.17 to 1.92)	0.001	1.45 (1.05 to 2.01)	0.025	1.26 (0.88 to 1.80)	0.214
High	1.02 (0.77 to 1.59)	0.577	0.93 (0.57 to 1.51)	0.760	0.72 (0.41 to 1.26)	0.251
Age at initiation of current drug						
<20	1.00		1.00		1.00	
20-29	1.11 (0.78 to 1.57)	0.579	0.75 (0.49 to 1.14)	0.176	1.03 (0.64 to 1.66)	0.891
≥30	2.11 (1.40 to 3.20)	<0.001	1.14 (0.67 to 1.91)	0.632	1.92 (1.07 to 3.47)	0.030
Duration of the current drug use						
<2.9	1.00		1.00		1.00	
3-9.9	1.25 (0.94 to 1.68)	0.130	1.27 (0.87 to 1.86)	0.218	2.34 (1.52 to 3.60)	<0.001
≥10	1.65 (1.19 to 2.29)	0.002	1.49 (0.96 to 2.3)	0.072	2.31 (1.40 to 3.81)	0.001

^a^The use of soft opioids was set as the baseline level. Goodness of fit indicators=Likelihood Ratio Test: *χ*^2^*(df*=33) = 211.03, *P* value ≤0.001. Pseudo R square= 0.12.
